# RNASeq data from Indonesian recalcitrant and non-recalcitrant rice varieties on anther culture

**DOI:** 10.1016/j.dib.2022.108760

**Published:** 2022-11-17

**Authors:** Siti Fatimah, Mohammad Syafii, Siti Zulaeha, Mega Dewi Haristianita, Devit Purwoko

**Affiliations:** a Agrotechnology Study Program, Faculty of Agriculture, Trunojoyo University, Unijoyo Campus, Telang, PO. BOX 2 Kamal – Bangkalan, East Java, Indonesia; b Research Center for Genetic Engineering, Research Organization for Life Sciences and Environment, National Research and Innovation Agency, Building No. 630 PUSPIPTEK South Tangerang Banten 15314, Indonesia; c The University of Tokyo 7 Chome-3-1 Hongo, Bunkyo City, Tokyo 113-8654, Jepang

**Keywords:** *Oryza sativa*, Anther culture, Dihaploid, Recalcitrant, Transcriptomics

## Abstract

The assembly of dihaploid rice plants through anther culture was constrained due to the recalcitrant properties. A comprehensive investigation of gene expression patterns among rice varieties with recalcitrant and non-recalcitrant anthers will help to understand the cellular mechanisms and biological processes of recalcitrant properties in rice anther cultures. Therefore, we performed RNA sequencing and analysis on the anthers of three selected Indonesian rice varieties with opposite recalcitrant properties. The varieties are Fatmawati with non-recalcitrant properties, IR64 recalcitrant and Tarabas unknown. The Illumina NextSeq PE150 sequencer was used to generate a total crude nucleotide of approximately 41.21 Gb in size. From 272,239,682 total paired final raw reads, 137,343,391 total net reads were obtained and uploaded to NCBI's Sequence Read Archive (SRA) repository under BioProject accession number PRJNA856048. This dataset allowed us to identify and profile all expressed genes with functions associated with recalcitrant and non-recalcitrant properties. In addition, the transcriptome data obtained will be valuable for the discovery of potential gene markers and functional SNPs associated with functional traits to assist rice breeding programs through the development of Marker Assisted Selection (MAS).

**Specifications Table**

**Table:** 

Subject	Agricultural and Biological Sciences
Specific subject area	Plant Transcriptomics
Type of data	Table, text file
How the data were acquired	Illumina NextSeq PE150 platform.
Data format	Raw (FASTQ)
Description of data collection	The anthers of three rice plants (Fatmawati, IR64 and Tarabas) were taken from young tassels when the microspore stage was in the uninucleate stage [1]. RNA of three rice anther samples was extracted using GeneAll® Ribospin™ Plant (GeneAll Biotechnology Co., Ltd.) and was submitted for RNA sequencing.
Data source location	Rice anther samples were collected at: • Institution: Trunojoyo University • City/Town/Region: Telang, Bangkalan, Madura • Country: Indonesia
Data accessibility	Repository name: NCBI Sequence Read Archive (SRA) Data identification number: PRJNA856048 Direct URL to data: https://www.ncbi.nlm.nih.gov/bioproject/PRJNA856048 The sequencing reads of three rice anther set are available in NCBI SRA accession number SRX16357934 (Tarabas), SRX16357933 (IR64), SRX16357932 (Fatmawati). https://www.ncbi.nlm.nih.gov/sra/SRX16357934 https://www.ncbi.nlm.nih.gov/sra/SRX16357933 https://www.ncbi.nlm.nih.gov/sra/SRX16357932 Mendeley Data Data identification number (permanent identifier, i.e. DOI number): 10.17632/w5nj767fzk.2 Direct link to the dataset: https://data.mendeley.com/datasets/w5nj767fzk[2]

**Value of the Data**

• These transcriptome data from young tassels at the uninucleate stage of the microspore stage were generated from the selected 3 rice varieties, which represent recalcitrant and non-recalcitrant complete sets of transcriptome data.
• Using this information, we can identify genes that are useful for understanding the molecular and cellular underpinnings of recalcitrant traits in rice anther cultures.
• With the help of these data, rice anther culture recalcitrant and non-recalcitrant transcriptomics can be compared. Different gene expression levels between types might aid in understanding the biological and molecular mechanisms behind some important rice features.
• Future genetic improvement studies on dihaploid rice will use these RNAseq data in conjunction with rice genome data to identify functional markers, such as single nucleotide polymorphisms (SNPs) and microsatellites related with recalcitrant traits.

## 1 Data Description

Anther culture is one of the in vitro techniques to accelerate the acquisition of pure lines in the form of androgenic dihaploid (DH) plants [3]. The dataset in this article is RNA-seq raw reads for anther taken from young tassels when the microspore stage was in the uninucleate stage of 3 rice varieties (Fatmawati with non-recalcitrant properties (F1), IR64 recalcitrant and Tarabas unknown). The raw data obtained from the Illumina NextSeq PE150 sequencer were deposited as a FASTQ format in NCBI's Sequence Read Archive (SRA) repository under BioProject accession number PRJNA856048. Accession numbers for each rice variety in the Mandeley database are presented in Table 1. Sequencing data analysis of each rice variety e.g. raw and clean reads, raw and clean nucleotides were performed as shown in Table 2. The quality of the net reads was assessed and a high quality percentage of net reads were obtained. The high quality reads were assembled to generate the contigs and mapped to Oryza_sativa.IRGSP-1.0 reference genome, the number of mapped contigs was estimated (Table 3). Oryza_sativa.IRGSP-1.0 reference genome was used for contigsmapping as it is a well-assembled and annotated genome, whereas the genome of indica rice cultivars has not yet been properly annotated [4]. In addition, transcript assembly for the reference genome with an ORF minimum 400 bp predicted the number of transcripts for each rice variety as listed in Table 3.

**Table 1:** Shows the summary of sequence information including the rice variety, phenotype, and fastq ID assigned to the metadata.

Rice variety	Phenotype	Fastq ID
IR-3	recalcitrant	IR-3_S7_L001_R1_001.fastq
		IR-3_S7_L001_R2_001.fastq
		IR-3_S7_L002_R1_001.fastq
		IR-3_S7_L002_R2_001.fastq
		IR-3_S7_L003_R1_001.fastq
		IR-3_S7_L003_R2_001.fastq
		IR-3_S7_L004_R1_001.fastq
		IR-3_S7_L004_R2_001.fastq
F1	non-recalcitrant	F1_S8_L001_R1_001.fastq
		F1_S8_L001_R2_001.fastq
		F1_S8_L002_R1_001.fastq
		F1_S8_L002_R2_001.fastq
		F1_S8_L003_R1_001.fastq
		F1_S8_L003_R2_001.fastq
		F1_S8_L004_R1_001.fastq
		F1_S8_L004_R2_001.fastq
RTR3	unknown	RTR-3_S9_L001_R1_001.fastq
		RTR-3_S9_L001_R2_001.fastq
		RTR-3_S9_L002_R1_001.fastq
		RTR-3_S9_L002_R2_001.fastq
		RTR-3_S9_L003_R1_001.fastq
		RTR-3_S9_L003_R2_001.fastq
		RTR-3_S9_L004_R1_001.fastq
		RTR-3_S9_L004_R2_001.fastq

**Table 2:** Statistics of sequencing data of individual recalcitrant and non-recalcitrant rice variety.

Rice variety	Phenotype	Raw reads (paired-end)	Raw nucleotides (bp)	Clean reads (paired-end)	Clean nucleotides (bp)
IR-3	recalcitrant	52,150,394	14,132,155,236	47,404,708	13,066,022,103
F1	non-recalcitrant	41,127,935	11,220,340,364	37,385,293	10,290,236,364
RTR3	unknown	44,065,061	12,189,584,927	40,055,141	11,232,417,836
Total		137,343,391	37,542,080,527	124,845,142	34,588,676,303

**Table 3:** Statistics of contigs assembling, contigs mapping and number of ORF for each recalcitrant and non-recalcitrant rice variety.

Rice variety	High-quality reads (paired-end)	Percentage of high quality reads (%)	Contigs Assembled	Mapped contigs	Percentages of mapped contigs (%)	Number of ORF
IR-3	47404708	90.9	98207	22483	22.89	88376
F1	37385293	90.9	92226	17690	19.18	83463
RTR3	40055141	90.9	91010	17659	19.40	84307
Total	124845142		281443	57832		256146

## 2 Experimental Design, Materials, and Methods

### 2.1 Sample sites taken

Rice was grown in the greenhouse at Trunojoyo University in Madura, East Java, Indonesia, in pots filled with soil from rice fields. Each pot contained four plants. The fertilization procedure involved the use of three different fertilizers: urea at a dose of 200 kg ha-1 (5 g/pot), SP36 at a dose of 100 kg ha-1 (2.5 g/pot), and KCl at a dose of 100 kg ha-1 (2.5 g/pot). Maintenance is carried out based on the lowland rice crop.

### 2.2 Anther sampling and RNA extraction

The pregnant stage was used to harvest the rice panicles of Tarabas, IR64, and Fatmawati. As part of the cold temperature pre-treatment, panicles were cleaned, wrapped in paper towels wet with water, placed in a zip-lock plastic bag, and kept at low temperatures for seven days [1]. Additionally, panicles were chosen so that the anthers would be in the uninucleate microspore growth stage when they were used as samples. The chosen anthers were then placed in liquid nitrogen to freeze, and they were kept at 80°C for later use. Following the technique, the total RNA was extracted using RibospinTM Plant (GeneAll, 2012). Then, using the RNA Nano 6000 kit in the Bioanalyzer 2100 system, the quantity and quality of RNA were assessed (Agilent Technologies, CA,).

### 2.3 Library preparation and next-generation sequencing

The mRNA library was purified using the TruSeq RNA sample preparation v2 kit (Illumina Inc, CA, USA) according to the manufacturer's protocol. Literature quantification was carried out using a Qubit Fluorometer and standard real-time PCR. The libraries were then sequenced using the Illumina NovaSeq 6000 PE150 (Novogene, China).

### 2.4 Data analysis

Initially, FASTQC was used to do quality control on the produced reads [5]. Trimmomatic was used to filter the raw reads to get rid of any low-quality reads (v0.39, Bolger et al 2014). From raw reads, data filtering involves eliminating adaptor sequences, contaminants, and poor-quality reads. The transcriptome was assembled using rnaSPAdes 3.15.3 in Galaxy at type of paired end: default (–pe); orientation of reads: FR (-><-); an additional set of short-reads: disabled; k-mer detection option: auto; Phred quality offset: auto; strand specificity: disabled and a minimum length of 150 bp using high-quality reads [6]. The contigs were mapped onto the reference genome using geneious RNA at medium sensitivity, executing five iterations [7]. The analyses mentioned above used the default parameters. Further downstream analyses, such as calling SNPs, co-expression networks for genes, and differentially expressed gene analysis, will utilise these sequences and information [8].

## 3 Ethics Statements

The author hereby consciously assures that for the manuscript **RNASeq data from Indonesian Recalcitrant and Non-recalcitrant rice varieties on anther culture** the following elements are fulfilled:

(1) It is the authors' original work, which has not been published anywhere else.
(2) This document is not being published anywhere else.
(3) The document reflects the author's research and analysis in a truthful and comprehensive way.
(4) The paper rightly assigns significant contributions from co-authors and co-researchers.
(5) All sources used are correctly disclosed (good citation).
(6) All authors have been personally and actively engaged in substantive work leading up to the document, and will take public responsibility for its content.

I agree with the above statements and declare that this submission complies with the Data In Brief policies contained in the Author's Guide and the Statement of Ethics [9].

## CRediT authorship contribution statement

**Siti Fatimah:** Project administration, Supervision, Writing – review & editing. **Mohammad Syafii:** Conceptualization, Methodology, Writing – original draft. **Siti Zulaeha:** Data curation, Resources, Investigation. **Mega Dewi Haristianita:** Supervision, Writing – review & editing. **Devit Purwoko:** Data curation.

## Declaration of Competing Interest

The authors declare that they have no known competing financial interests or personal relationships that could have appeared to influence the work reported in this paper.

## Data Availability

RNASeq data profile from Indonesian Recalcitrant and Non-recalcitrant rice varieties on anther culture (Original data) (Mendeley Data).

## References

[1] Dash B., Bhuyan S.S., Singh S.K., Chandravani M., Swain N., Rout P., Katara J.L., Parameswaran C., Devanna B.N., Samantaray S. (2022). Androgenesis in *indica* rice: A comparative competency in development of doubled haploids. PLOS One.

[2] Purwoko Devit, Zulaeha Siti, Fatimah Siti, Syafii Mohammad, Haristianita Mega Dewi (2022). “RNASeq data profile from Indonesian Recalcitrant and Non-recalcitrant rice varieties on anther culture”. Mendeley Data.

[3] Dewi I.S., Putri N.H., Purwoko B.S. (2019). Response of Anther Donor Genotypes (F1) from *Indica* x *Indica* Crosses to Rice Anther Culture. Jurnal AgroBiogen.

[4] Tanaka T., Nishijima R., Teramoto S., Kitomi Y., Hayashi T., Uga Y., Kawakatsu T. (2020). *De novo* Genome Assembly of the *indica* Rice Variety IR64 Using Linked-Read Sequencing and Nanopore Sequencing. G3 Genes|Genomes|Genetics.

[5] S. Andrew. FastQC; a quality control tool for high throughput sequence data (2010) Available online at https://www.bioinformatics.babraham.ac.uk/projects/fastqc/.

[6] Bushmanova E., Antipov D., Lapidus A., Prjibelski A.D. (2019). (2018). rnaSPAdes: a de novo transcriptome assembler and its application to RNA-Seq data. GigaScience.

[7] Geneious Prime 2022.1.1. (http://www.geneious.com).

[8] Zainal-Abidin R.A., Zainal Z., Mohamed-Hussein Z.A., Abu-Bakar N., Ab Razak M.S.F., Simoh S., Sew Y.S. (2020). RNA-seq data from whole rice grains of pigmented and non-pigmented Malaysian rice varieties. Data Brief..

[9] Purwoko D., Safarrida A., Tajuddin T., Rupaedah B., Suyono A., Wahid A., Sugianto M., Suja'i I. (2022). Metagenomic data of microbial in natural empty fruit bunches degradation. Data Brief.

